# Management of Non-Tubal Ectopic Pregnancies: A Single Center Experience

**DOI:** 10.3390/diagnostics10090652

**Published:** 2020-08-31

**Authors:** Guglielmo Stabile, Giulia Zinicola, Federico Romano, Francesca Buonomo, Francesco Paolo Mangino, Giuseppe Ricci

**Affiliations:** 1 Institute for Maternal and Child Health IRCCS “Burlo Garofolo”, 34137 Trieste, Italy; federico.romano@burlo.trieste.it (F.R.); francesca.buonomo@burlo.trieste.it (F.B.); francesco.mangino@burlo.trieste.it (F.P.M.); giuseppe.ricci@burlo.trieste.it (G.R.); 2 Department of Medicine, Surgery and Health Sciences, University of Trieste, 34127 Trieste, Italy; giulia.zinicola@burlo.trieste.it

**Keywords:** ectopic non-tubal pregnancy, hysteroscopy, laparoscopy, fertility sparing

## Abstract

Non-tubal ectopic pregnancies (NT-EPs) are rare but potentially life-threatening conditions. The incidence ranges are between 5–8.3% of all ectopic pregnancies. For this retrospective observational study, 16 patients with NT-EP and treated from January 2014 to May 2020 were recruited. Demographic details, symptoms, Beta human chorionic gonadotrophin (β-hCG) levels, ultrasound findings, management and treatment outcomes were presented. In hemodynamically stable patients, diagnosis was made using ultrasounds and β-hCG levels. Laparoscopy was essential to identify and remove the ectopic pregnancy in clinical unstable patients. A radical laparoscopic approach was chosen in one case of cervical pregnancy diagnosed late in the first trimester. Medical treatment and minimally invasive procedure, alone or combined, resulted in effective strategies in asymptomatic women with an early diagnosis of NT-EP. We report cases of cervical pregnancies successfully treated by hysteroscopy alone or combined with medical treatment, the first case of scar pregnancy treated by mini-reseptoscope and curettage and the fifth case of interstitial pregnancy treated with Methotrexate and Mifepristone. In this manuscript we report a single center experience in the management of NT-EPs with the aim of outlining the importance of the early diagnosis for a minimally invasive treatment in order to reduce maternal morbidity and mortality and preserve future fertility.

## 1. Introduction

Ectopic pregnancies (EP) account for 2% of all pregnancies and in most of the cases gestational sac (GS) is implanted within the fallopian tube [1]. Pregnancy could be rarely revealed in other sites, such us cervix, ovary, abdomen, interstitial portion of the fallopian tube and cesarean scars. The incidence range of non-tubal EP (NT-EP) is between 5% and 8.3% of all EP, and it has increased in the last two decades with the widespread use of assisted reproductive techniques (ARTs) [1]. The frequency of cervical ectopic pregnancies (CPs) accounts for < 1% of all EPs [2], while Cesarean scar pregnancies (ScPs) and interstitial ectopic pregnancies (IPs) may represent up to 4.2% [3] and 2–11% of all EPs, respectively [3,4]. Some of the identifiable risk factors include genital tract infection; intrauterine devices; previous EP; Asherman′s Syndrome; endometriosis; tubal and uterine surgery, including tubal sterilization, myomectomy, cesarean section, uterine curettage; and smoke [5]. NT-EP may not be associated with tubal pathology [6]. Diagnosis involves a combination of variables. The most common symptom is vaginal bleeding, which is often profuse and painless. Serial β-hCG levels are commonly used to monitor early pregnancies, but the ultrasound findings of the GS are essential [7]. NT-EPs are rare but potentially life-threatening because they are often diagnosed when symptoms of rupture appear. Implantation site of the pregnancy affects the severity of the disease [2]. Early diagnosis and effective treatment are essential to reduce the immediate and delayed side effects, with a significant reduction of maternal morbidity and mortality. Advances in ultrasound technology and development of diagnostic tests increased the earlier diagnosis for NT-EP. NT-EP may be successfully treated conservatively if an early diagnosis occurs before clinical symptoms of rupture appear, performing a medical or minimally invasive surgical treatment in patients with desire for future pregnancies [6]. In this manuscript, we report a single-center experience in the management of NT-EPs with the aim of outlining and suggesting the best possible strategy for fertility sparing in hemodynamically stable patients. 

## 2. Materials and Methods

The institutional review board (RC 08/2020) approved this retrospective observational descriptive study in February 2020.

**Figure 1:**
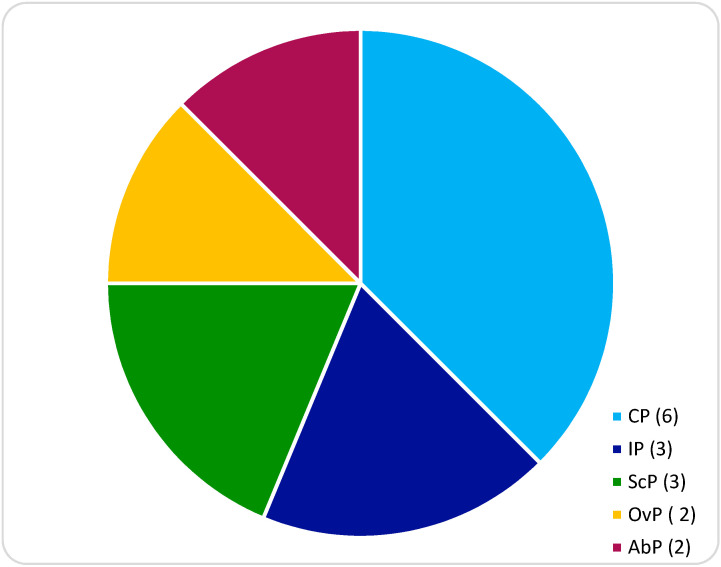
Non-tubal ectopic distribution by location in our center: Cervical pregnancy (CP); Interstitial pregnancy (IP); Scar pregnancy (ScP); Ovarian pregnancy (OvP); Abdominal pregnancy (AbP).

**Figure 2:**
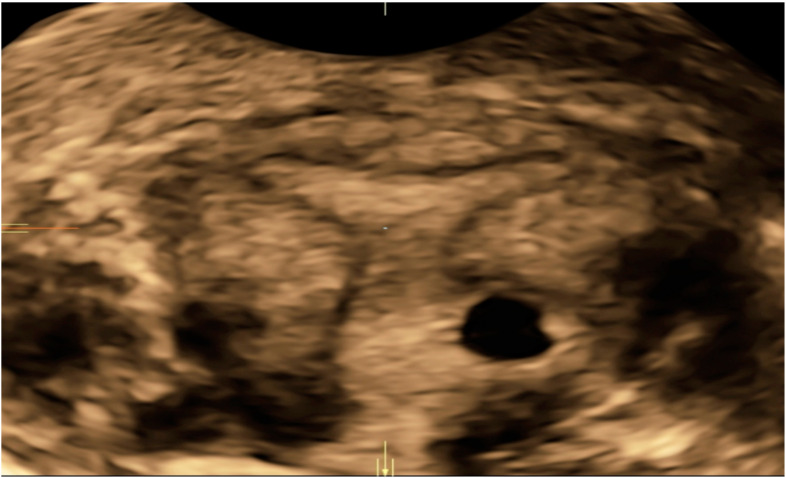
3D Volume contrasting imaging (VCI) image of a cervical ectopic pregnancy. No evidence of intrauterine pregnancy, hourglass shape of uterus, cervical ballooning, presence of placental tissue or gestational sac within the cervical canal and closed internal uterine orifice.

Patients with a diagnosis of NT-EP treated at the Institute of Child and Maternal Health Burlo-Garofolo in Trieste, Italy, from January 2014 to May 2020 were recruited. All patients have signed an informed consent before treatment. Permission for the publication was taken in accordance with the 1964 Helsinki Declaration and its later amendments or comparable ethical standards. Obstetrical/gynecological history, previous risk factors for ectopic non-tubal pregnancy, serum β-hCG levels at the diagnosis, ultrasounds findings, surgical or medical management and treatment outcomes are presented (Table 1 and Table 2). We identified *n* = 16 NT-EPs divided into four groups on the basis of the implantation site: cervical pregnancies (CP) (*n* = 6), interstitial pregnancies (IP) (*n* = 3), cesarean scar pregnancies (ScP) (*n* = 3), abdominal pregnancies (AbP) (*n* = 2), ovarian pregnancies (OvP) (*n* = 2) (Figure 1). Diagnosis of CP was made by transvaginal ultrasound (TVUS) according to criteria given by Hofmann et al. in 1987, and they consist of no evidence of intrauterine pregnancy, hourglass shape of uterus, cervical ballooning, presence of placental tissue or gestational sac within the cervical canal and closed internal uterine orifice [8]. The “sliding organ” sign was absent [8] (Figure 2).

**Figure 3:**
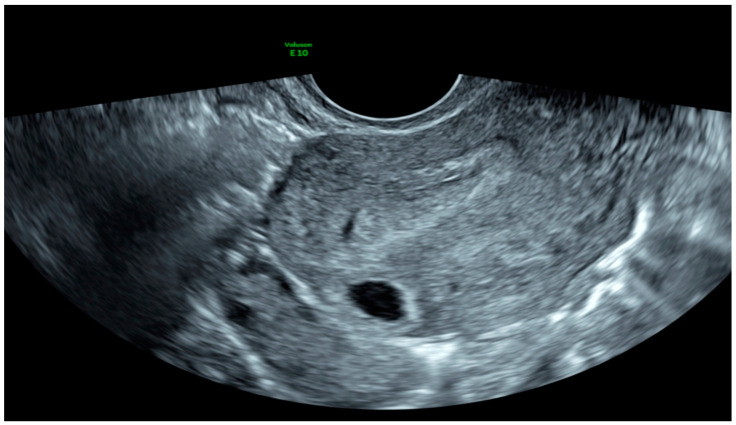
2D Transvaginal image of an interstitial ectopic pregnancy. The empty uterine cavity, the myometrial layer of less than 5 mm surrounding the GS, a chorionic sac separated and laterally located >1 cm from the sideward portion of the uterine cavity, the visualization of the interstitial line between the GS and the lateral edge of the endometrial cavity and the myometrial mantle around the ectopic GS.

Diagnosis of IP was made by TVUS according to criteria outlined by Timor-Tritsch that consist of an empty uterine cavity, a myometrial layer of less than 5 mm surrounding the GS and a chorionic sac separated and laterally located 1 cm or more from the sideward portion of the uterine cavity [9]. Moreover, the visualization of the interstitial line between the GS and the lateral edge of the endometrial cavity and the myometrial mantle around the ectopic sac helped for diagnosis [10] (Figure 3).

**Figure 4:**
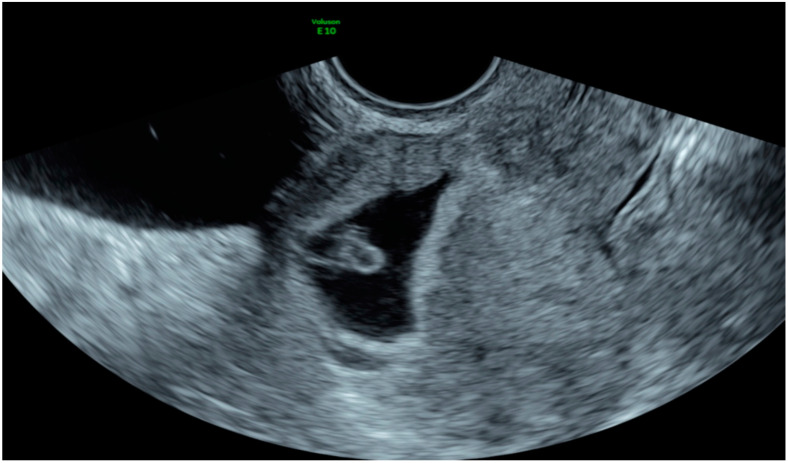
2D Transvaginal image of a scar pregnancy. No fetal parts in the uterus or cervix, the presence of a GS in the anterior isthmic portion covering the scar site, entirely embedded within the myometrium and absence of contact between the bladder and GS.

Criteria used for the diagnosis of ScP included no fetal parts in the uterus or cervix, the presence of a GS in the anterior isthmic portion covering the scar site or entirely embedded within the myometrium and absence of contact between the bladder and GS [11] (Figure 4). 

**Figure 5:**
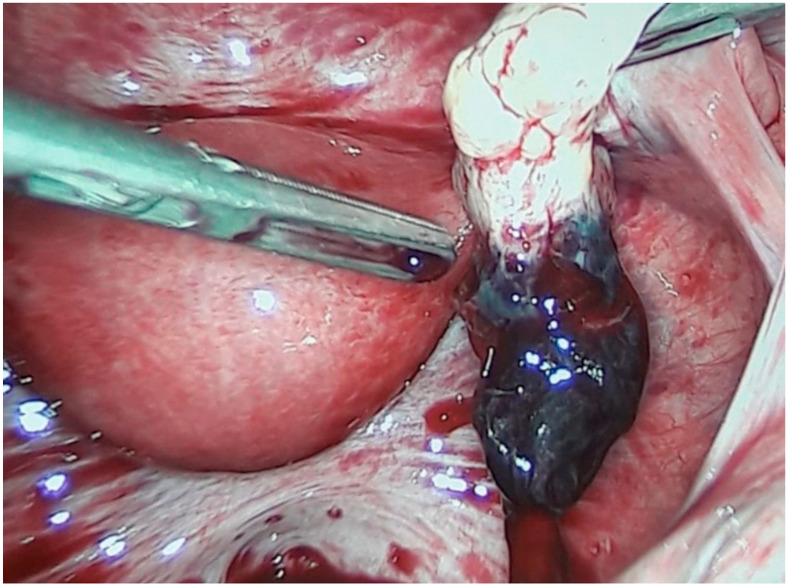
Laparoscopic image of an ovarian ectopic pregnancy.

Abdominal and Ovarian pregnancy (Figure 5) were detached and treated during laparoscopy in patients with high serum β-hCG levels and hemoperitoneum.

A fertility sparing treatment was administrated in five cases of CP: A total medical management using a single dose of MTX IM 50 mg/m^2^ of the body surface in addition to Mifepristone 600 mg and Misoprostol 400 mcg orally (*n* = 1), a combined treatment using a previous single dose of MTX IM injection at dosage of 50 mg/m^2^ of the body surface followed by hysteroscopy (*n* = 2) and a totally hysteroscopic approach (*n* = 2). One case of CP was diagnosed late in the first trimester, and it was treated by laparoscopy. 

Conservative medical management for IPs involved the use of MTX (*n* = 1), the combination of systemic MTX with Mifepristone 600 mg (*n* = 2).

**Figure 6:**
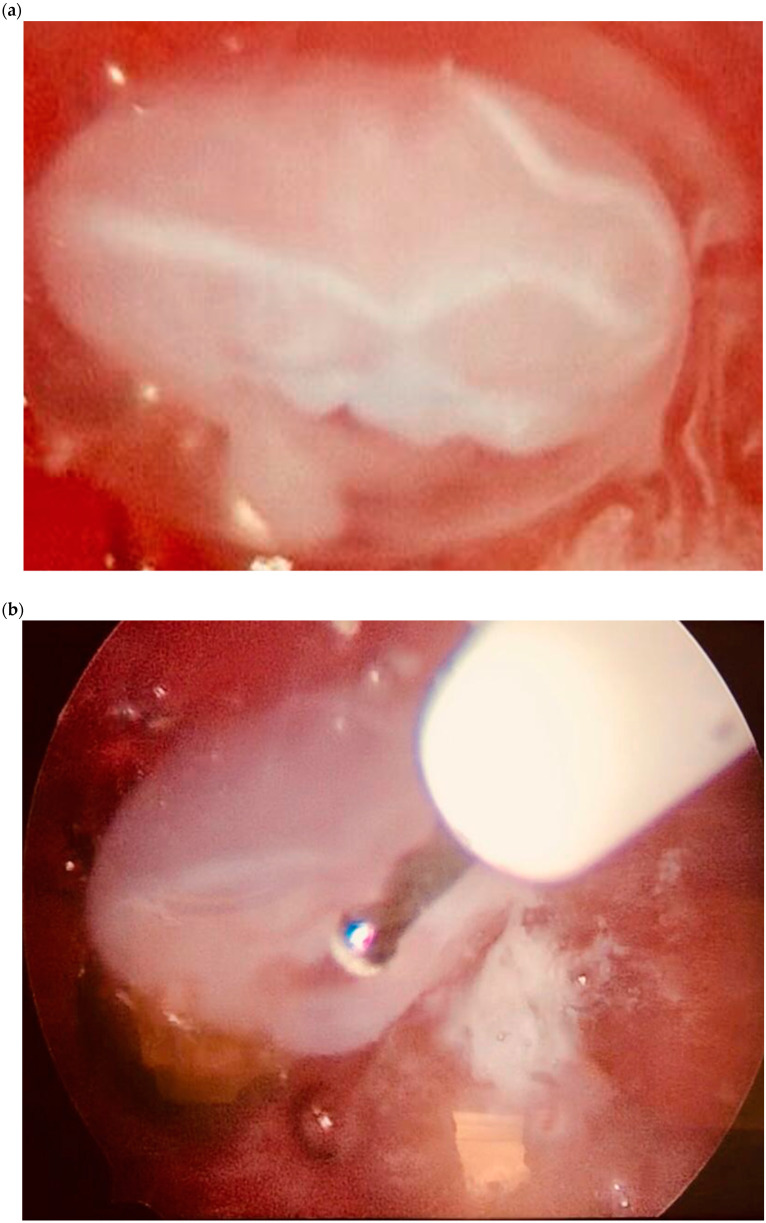

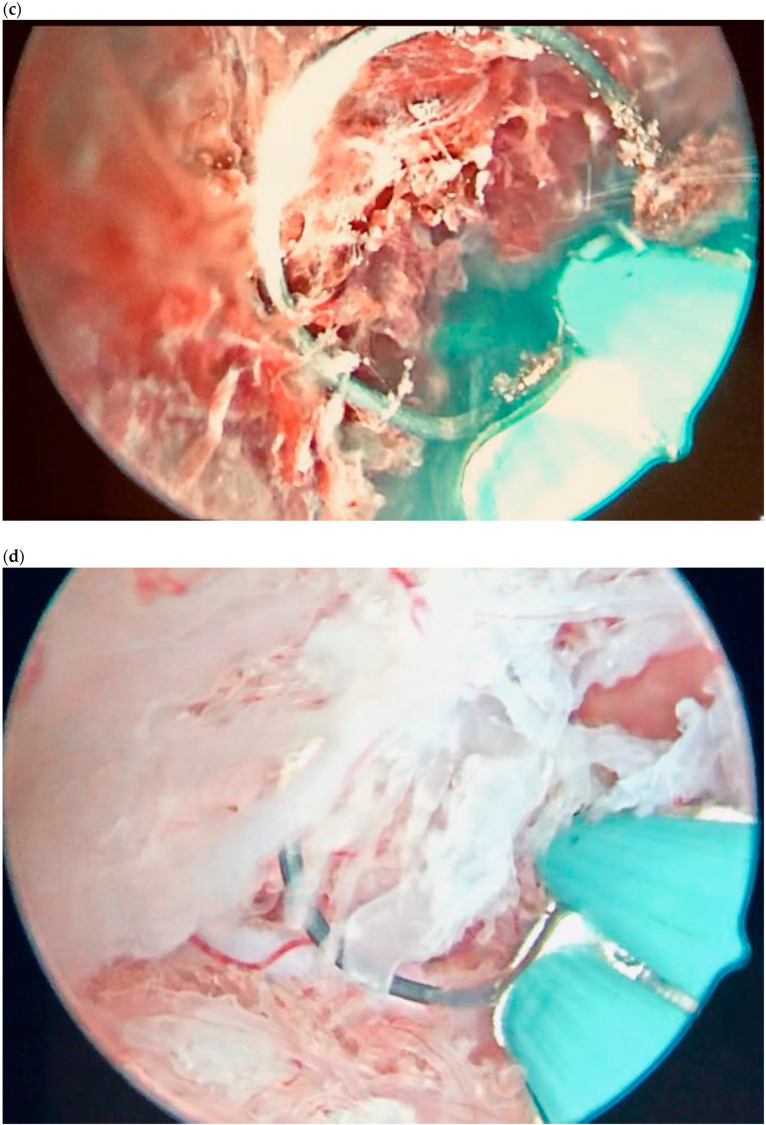
Hysteroscopic view of a scar pregnancy. (**a**) Embryo view. (**b**) Embryo coagulation by a bipolar electrode. (**c**) Resection of the trophoblast and its detachment from the myometrium. (**d**) Resection of the trophoblast and its detachment from the myometrium.

**Figure 7:**
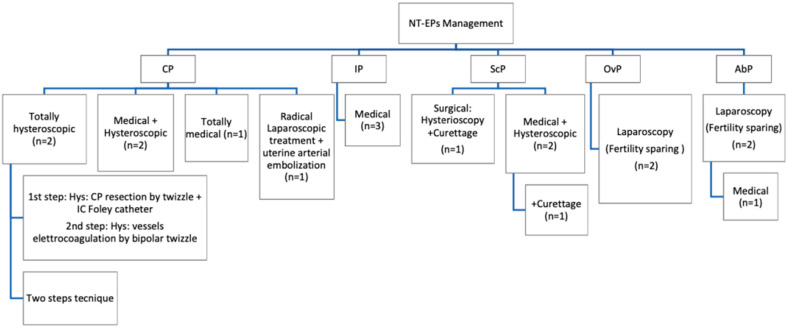
Overview of managment for each type of non-tubal ectopic pregnancy. CP: the medical (*n* = 1), hysteroscopic (*n* = 2) or the combined approach (*n* = 2) were administrated; only in one of the cases, a radical surgical intervention with uterine arterial embolization was necessary. IP: A totally medical treatment resulted sufficient (*n* = 2). ScP: A minimally invasive approach (hysteroscopy and curettage) resulted effective in one case of ScP; in *n* = 2 patient, a combined approach was chosen (medical and hysteroscopic), and in one of these cases, it was necessary to manage the uterine curettage. OvPs and AbPs were treated by laparoscopy. In one of the cases of AbP, also a single systemic dose of MTX was administrated.

For ScPs, the conservative treatment provided the use of the hysteroscopic strategy after a single dose of systemic MTX (50 mg/m^2^ of the body surface combined to rectal Misoprostol (*n* = 1) or the combination of Mifepristone and Misoprostol (*n* = 1). Hysteroscopy alone was chosen in one case (*n* = 1) (Figure 6). Additionally, with the aim of remove completely trophoblastic residues, curettage had been administrated at the end of the hysteroscopic procedure (Table 2; Figure 7).

## 3. Results

During the study period, a total of *n* = 16 NT-EPs were listed in our institution’s records. Background and clinical characteristics of the study patients are summarized in Table 1. In hemodynamically stable patients, diagnosis was made on the basis of TVUS findings and serum β-hCG levels; in *n* = 4 cases, emergency laparoscopy was essential in order to identify and remove the EP in clinically not stable women with hemoperitoneum/acute abdomen symptoms. In particular, we identified *n* = 2 AbPs, *n* = 2 OvPs, *n* = 3 IPs, *n* = 3 ScPs, and *n* = 6 CPs (Figure 1). The mean age of the study cohort at diagnosis was 35 years (range 19–41). The mean GA at diagnosis was 6 + 3 weeks (range 12 + 1/5 weeks) as shown in Table 1. *n* = 2 pregnancies were achieved by ARTs; the others were conceived spontaneously. Among known risk factors, *n* = 5 women had almost a previous cesarean delivery (CD) (*n* = 1 IP; *n* = 1 CP; *n* = 3 ScP), *n* = 2 women had undergone a previous salpingectomy because of a history of tubal EP. Symptoms at diagnosis included abdominal pain and/or vaginal bleeding in *n* = 8 patients; hemoperitoneum and abdominal acute pain was recorded in *n* = 4 cases of OvPs and AbPs; no symptoms were recorded in *n* = 4 cases (Table 1). 

Level of β-hCG by treatment group are presented in Table 2. 

Among primary treatments, *n* = 4 women were managed conservatively solely by medical treatment. Because of the failure of initial medical treatment, *n* = 5 women required for surgery. In *n* = 1 case of AbP, the laparoscopic approach was combined with the systemic administration of MTX IM 50 mg/m^2^ of the body surface. A laparoscopic fertility sparing approach has been used in hemodynamically unstable women with OvPs (*n* = 2) and AbPs (*n* = 2). In *n* = 1 case of CP, the diagnosis had been made late in the first trimester because of the delayed discovery of the pregnancy status: the patient was totally asymptomatic with a cervical GS with a diameter of 78 × 60 mm. The radical laparoscopic approach with a total hysterectomy combined with uterine arterial embolization had been chosen in order to reduce the hemorrhagic risk. 

Surgeons had decided for a conservative treatment using hysteroscopy, alone or in combination to medical treatment, in hemodynamically stables patients (*n* = 7) with a diagnosis of CP and ScP at an early GA and low serum β-hCG level in order to preserve future fertility. Additionally, after the hysteroscopic procedure, curettage had been made in the group of ScPs disease. A detailed description of primary and secondary treatments performed is shown in Table 2 and Figure 7. 

## 4. Discussion

NT-EPs represent an important challenge for the gynecologist because of the rarity of the disease and the lack of guidelines for its management. The risk of EP following in vitro fertilization (IVF) has been estimated as high as 2–5% [12], with the consequent increased incidence of heterotopic pregnancies.

With the widespread availability and application of ultrasound, identifying earlier the location of the gestational sac has become possible, guiding the gynecologist in choosing how to manage ectopic pregnancies appropriately [13].

In order to reduce morbidity and mortality related to a delayed diagnosis and the appearance of life-threatening complications, it might be useful to refer patients to centers of excellence. The availability of dedicated early pregnancy units has improved diagnosis and the follow-up for these patients [14].

The management of each patient must be individualized based on clinical symptoms, viability of pregnancy, GA, hCG levels and women’s wishes.

The acquisition of expertise in the care of NT-EPs ensures a faster diagnosis, which gives a better chance of successful medical therapy or minimally invasive surgery, such as the local injection of one of several agents MTX, potassium chloride (KCl), hyperosmolar glucose, etoposide (directly into the GS [15]), curettage, hysteroscopy or laparoscopy in order to make feasible a fertility sparing approach in most of the cases.

We have presented data from a six-year review of all diagnosed NT-EPs and their subsequent management. This series adds to the growing body of evidence that the hysteroscopic approach, combined with systemic MTX or alone, is a safe and efficacious first-line treatment for women with high-risk NT-EPs desiring to preserve future fertility.

In general, the primary treatment option for most IP and OvP is surgery; medical treatment with systemic MTX (50 mg/m^2^ body surface area) or local MTX (1 mg/kg body weight)) is preferred for ScP and CP. Expectant management is possible in asymptomatic patients with nonviable pregnancy and decreasing hCG levels. Women with heavy bleeding or failed medical treatment need surgical procedures [16].

Surgical laparoscopic management is indicated in women with contraindications to medical treatment, hemodynamic compromise or other clinical signs of ruptured NT-EP including abdominal pain or evidence of intra-abdominal bleeding and according to patient preference [17]. The standard surgical intervention had been laparotomy until the laparoscopic approach was introduced in 1973 by Shapiro and Adler, and the latter has gained wide acceptance. Three prospective randomized trials have demonstrated the superiority of a laparoscopic approach over laparotomy in terms of lower blood loss, pain medication requirement, length of hospital stay and costs. Reproductive outcomes, including rates of recurrent EP and subsequent intrauterine pregnancy, are not significantly different in the two groups [17]. Contralateral fallopian tube status and desire for future fertility have to be taken into consideration when a surgical approach is chosen. In recent years, laparoscopy was thought to be a minimally invasive surgical procedure, which could better protect normal ovarian tissue and reduce pelvic adhesion [18]. These features satisfy the main objectivity of conservative surgery: to guarantee patient safety.

### 4.1. Cervical Pregnancy

CP has a reported incidence of 1 in 1000–18,000 pregnancies. It is considered that there is a high risk of hemorrhage as a consequence of CP, and CP has historically been treated with hysterectomy, leading to loss of fertility [19]. Sonographic diagnostic criteria reported by Jurkovic et al. are (1) empty uterine cavity or thickened endometrium, (2) distended and/or enlarged cervix, (3) GS or placental tissue below the level of the internal os, (4) negative “sliding organs sign” and (5) high peritrophoblastic vascularity on Doppler examination (peak velocity > 20 cm/s, pulsatility index < 1.0) [20].

With improvements in ultrasound, early diagnosis of CP has become possible with the subsequent possibility of conservative management.

For CP, we suggest the hysteroscopic approach, alone or combined with systemic MTX, also in cases of patients with β-hCG serum levels higher than 5000 UI/mL [21]. In case of local injection of MTX, hysteroscopy appears advantageous in comparison to ultrasonography. The hysteroscopic approach is a safer, faster and more accurate technique in comparison with other methods such as curettage, since direct visualization provides a precise resection and coagulation of the ectopic tissue, achieving complete eradication with minimal bleeding [22].

### 4.2. Interstitial Pregnancy

IP is a rare form of EP that usually leads to uterine rupture, generally at advanced gestational ages.

It is a life-threatening condition with a mortality rate 6–7 times higher. Quantitative β-hCG levels and TVUS are essential in order to manage this condition safely. An empty uterine cavity, a separate chorionic sac at least 1 cm from the lateral edge of the uterine cavity, the paucity of the myometrium around the gestational sac (<5 mm) and the interstitial line are diagnostic of IP [23].

Early diagnosis with TVUS allows conservative treatment with methotrexate; if it is made later in gestation, surgical treatment can be required.

From our analysis it emerged that medical management with MTX IM regimen isolated or combined with mifepristone (600 mg orally administered) can be considered a good option in women with IP and a strong motivation for future conceptions [24].

### 4.3. Scar Pregnancy

This complication is likely to become more common with the increased rates of abdominal delivery increasing worldwide and in 72% of cases occurs in women who have had more than 2 CDs [25,26].

TVUS is likely to emerge as a future gold standard for the diagnosis of scar implantation. Diagnosis is relatively simple in early pregnancy, but as the pregnancy progresses, the distinction between ScP, CP and low intrauterine pregnancy becomes more difficult. In women with non-viable pregnancies, Doppler ultrasound and “sliding organs sign” should be used to confirm the diagnosis of a scar pregnancy [27].

A range of treatment options are available to treat ScP; however, it is not clear which is the best option. Surgical procedures, alone or in combination with medical treatment, have high success rates, but greater surgical skill is required. Medical management is not considered treatment of choice for ScP probably because the absorption and efficacy of MTX is reduced by the fibrous tissue surrounding the GS, which is located in an unusual site inside the uterine cavity. When combined with curettage or hysteroscopy, MTX appears more efficient [28]. From a recent intervention review, it emerged that it is uncertain whether there is a difference in treatment success rates, complications, adverse effects or time to normalize β-hCG between suction curettage under hysteroscopy and under ultrasonography (very low-quality evidence) [29].

We have recorded 3 ScPs successfully treated by hysteroscopy, followed by curettage in one of the cases, in order to remove trophoblastic residues and reduce risk of subsequent reintervention. This minimally invasive approach allows the direct visualization of the implantation site and the possibility to separate the gestational sac from the myometrium under operator view. In literature, a success rate for this procedure of 8/8 is reported [26]. Hysteroscopic removal of ScPs has as advantages the faster normalization of β-hCG levels, the rapid return to normal morphology of the uterine cavity, the shorter follow-up and a faster return to fertility. In two of the cases, surgery had been combined with medical therapy. In one case, we used a two-step hysteroscopic technique for the first time ever, using in a first time the Twizzle electrode and in a second time the miniseptor. Essential criteria for conservative treatment remain the early diagnosis of the disease and the absence of clinical signs of hemodynamic instability.

### 4.4. Ovarian Pregnancy and Abdominal Pregnancy

OvP is a rare event, with estimates of frequency ranging from 1 in 2100 to 1 in 7000 pregnancies or 3% of all EPs.

A more echogenic wide ring on the ovary, compared with the ovarian tissue, a yolk sac or fetal parts are ultrasonographic findings for OvP. Surgical criteria where described by Spiegelberg: the fallopian tubes intact and separate from the ovary, the GS on the ovary must be attached to the uterus through the utero-ovarian ligament and the placental tissue appearing mixed with ovarian cortex. Surgical treatment is the most frequent approach, and an oophorectomy or a wedge resection of the ovary is usually required [30].

Abdominal pregnancy is defined as pregnancy anywhere within the peritoneal cavity and represents around 1–1.5% of all EPs with an estimated incidence of 1:8000–10,000 pregnancies. Maternal mortality is estimated to be 2–30%, while perinatal mortality in those undiagnosed is 40–95%.

Studdiford′s criteria are normal bilateral fallopian tubes and ovaries, the absence of utero-peritoneal fistula and pregnancy related exclusively to the peritoneal surface and early enough to eliminate the possibility of secondary implantation following a primary location in the tube.

TVUS is the first-line tool for diagnosis, but also, MRI could be considered in cases of ambiguity, especially to define the extent of the placental tissue invasion to the abdominal and pelvic organs. When images are inconclusive or patients become hemodynamical instable, laparoscopy is essential. Laparoscopic surgery should be considered for early abdominal pregnancy to allow better access to deal with placental attachment and control the bleeding [31].

According to the present literature, we report four cases enhanced with clinical symptoms of hemodynamical instability and acute abdomen and TVUS findings suggestive for OvPs and AbPs treated successfully with laparoscopy.

The main limitations of this manuscript are small sample sizes due to the rarity of the disease and heterogeneity in treatments on the basis of clinical specific characteristics and with the aim of individualizing the optimum mode of management.

It is imperative for a reference center for NT-EP management having well trained surgeons in minimally invasive surgery with specific skills, reducing risks of life-threating hemorrhage and hysterectomy and preserving future fertility.

## 5. Conclusions

From our sample emerges the necessity of a fast diagnosis of NT-EP. This can help a conservative management with a medical or minimally invasive approach. The important progress in imaging technologies allows a faster diagnosis, permitting the transfer of the patient to a reference center where the choice of the best procedures may reduce the impact of surgery on the patient’s fertility.

## Figures and Tables

**Table 1:** Demographic characteristics, obstetric and gynecological history and ectopic pregnancy risk factors in the study population.

Ectopic Pregnancy	Age	Obstetric and Gynecological History	Gravidity(g); Parity (p)	Pregnancy Onset
**Cervical Pregnancy**				
**Case 1**	37	2 previous cesarean sections 1 previous spontaneous abortion with curettage	3 g 2 p	Spontaneous
**Case 2**	35	Untreated sub-sept uterus Tubal pregnancy and salpingectomy	2 g 0 p	Spontaneous
**Case 3**	37	Infertility Mild uterine sub-sept	1 g	ARTs
**Case 4**	41	Cervical pregnancy treated with Methotrexate Previous miscarriage	4 g 0 p	Spontaneous
**Case 5**	35	/	1 g	Spontaneous
**Case 6**		Previous miscarriage	2 g 0 p	ARTs
**Scar pregnancy**				
**Case 1**	32	5 Previous cesarean sections	6 g 5 p	Spontaneous
**Case 2**	33	3 previous cesarean sections	4 g 3 p	Spontaneous
**Case 3**	34	1 previous cesarean section	3 g 1 p	Spontaneous
**Interstitial pregnancy**				
**Case 1**	32	Metroplasty for septate uterus	3 g 0 p	Spontaneous
**Case 2**	32	2011 cesarean section 2016 right tubal pregnancy treated by laparoscopic salpingectomy	3 g 1 p	Spontaneous
**Case 3**	35	/	3 g 1 p	Spontaneous
**Ovarian pregnancy**				
**Case 1**	19	/	1 g	Spontaneous
**Case 2**	33	/	2 g 1 p	Spontaneous
**Abdominal pregnancy**				
**Case 1**	39	/	1 g	Spontaneous
**Case 2**	41	Uterine fibromatosis	1 g	Spontaneous

**Table 2:** Non-tubal ectopic pregnancies: A single center experience. For each subtype of NT-EP are reported clinical presentation, gestational age, basal β-hCG levels, ultrasound findings, treatment details (medical or surgical or medical combined with surgical) and outcome.

Ectopic Pregnancy	Outset	Gestational Age (Weeks)	β-hCG before Treatment	Ultrasound	Treatment	Outcome
**Cervical pregnancy**						
**Case 1**	Asymptomatic	12 + 1	97,388	GS 78 × 60 mm; with embryo	Radical surgical treatment (LPS hysterectomy + bilateral salpingectomy + uterine arterial embolization + bilateral ureteral stent placement)	Complete resolution
**Case 2**	Asymptomatic	6 + 6	10,862	GS 20 × 19 mm; with embryo	Hys: 1st step: CP resection by twizzle; IC Foley catheter 2nd step: vessels electrocoagulation by bipolar twizzle	Reoperation (hys) due to tissue residues and cervical laceration Complete resolution
**Case 3**	Asymptomatic	5	9747	GS 20 × 22 mm; no embryo	MTX IM 50 mg/m^2^ of body surface + Hys	Complete resolution Currently pregnant (PMA Homologous)
**Case 4**	Brownish vaginal discharge	6 + 6	55,951	GS 30 × 10 mm; no embryo	Hys	Complete resolution
**Case 5**	Vaginal bleeding	9	1331	GS 4.7 × 5 mm; no embryo	Mifepristone 600 mg orally + Misoprostol 400 mcg + MTX IM 50 mg/m^2^ of body surface	Complete resolution
**Case 6**	Vaginal bleeding	6	4274	GS 5.4 × 5 mm with embryo	MTX IM 50 mg/m^2^ of body surface + Hys: CP resection by twizzle	Complete resolution
**Scar pregnancy**						
**Case 1**	Asymptomatic	6	119,900	GS 14 × 16 mm	MTX IM 50 mg/m^2^ of body surface + Misoprostol 200 µg rectal + Hys (two times) + Curettage	Complete resolution
**Case 2**	Asymptomatic	6 + 2	31,647	GS 12 × 10 mm	Mifepristone 600 mg + Misoprostol 400 mcg + Hys: reseptoscopy	Complete resolution
**Case 3**	Asymptomatic	7 + 1	131,000	GS 9 × 10 mm	Hys: twizzle and minireseptor + Curettage	Complete resolution
**Interstitial pregnancy**						
**Case 1**	Brownish vaginal discharge and mild pelvic pain	7	18,048	GS 15 × 14 mm right horn	MTX IM 50 mg/m^2^ of body surface	Complete resolution
**Case 2**	Asymptomatic	6	6579	GS 6 × 8 mm; right horn	MTX IM 50 mg/m^2^ of body surface + Mifepristone 600 mcg	Complete resolution
**Case 3**	Pinkish vaginal discharge	5 + 3	2124	GS right 12 × 12 horn; N/A	MTX IM 50 mg/m^2^ of body surface + Mifepristone 600 mg	Complete resolution
**Ovarian pregnancy**						
**Case 1**	Haemoperitoneum	unknow	2495	GS 30 mm (maximum diameter); on the right ovary	Surgical treatment (LPS)	Complete resolution
**Case 2**	Haemoperitoneum	unknown	2185	GS 30 × 20 mm on the right ovary	Surgical treatment (LPS)	Complete resolution
**Abdominal pregnancy**						
**Case 1**	Acute abdomen	6 + 3	N/A	GS 20 mm at the right wall of the peritoneum	Surgical treatment (LPS) + MTX IM 50 mg/m^2^ of body surface	Complete resolution
**Case 2**	Haemoperitoneum	7 + 2	53,716	GS 30 mm located in the patch of the Douglas	Surgical treatment (LPS)	Complete resolution
